# Coronary Artery Dilation in Children with Febrile Exanthematous Illness without Criteria for Kawasaki Disease -An Enigmatic Disease

**DOI:** 10.36660/abc.20190730

**Published:** 2019-12

**Authors:** Vitor Coimbra Guerra

**Affiliations:** 1 The Hospital For Sick Children, Sickkids, Toronto - Canada; 2 University of Toronto, Toronto - Canada

**Keywords:** Doenças Cardiovasculares/ diagnosis, Coronary Artery, Kawasaki Disease, Fever, Exantemous, Echocardiography/ diagnostic imaging.

More than half a century has elapsed since Prof. Tomisaku Kawasaki’s did the first description of a unique disease. He saw the first 4-year-old patient with fever and rash in 1961…. At that time, he described the diagnosis “unknown”. The published paper title was *Infantile acute febrile mucocutaneous lymph node syndrome with specific desquamation of the fingers and toes. Clinical observation of 50 cases.* ^1^ From This “unknown diagnosis”, which now we call Kawasaki disease (KD) until the current era, this vasculitis of unknown cause became the leading cause of acquired heart disease among children in United States.^2^

Historically, the presence of coronary abnormalities was not noticed until patients died suddenly of cardiac complications. An angiographic study of 1100 patients showed coronary artery lesions in 24%, with aneurysms in 8% and a number of patients with stenoses and occlusions.^3^ As soon as the Coronary arteries became the key structure for risk stratification, treatment and outcome, extensive research and worldwide effort has been done targeting the correct diagnostic. Unfortunately, to make things more challenging, there are forms of “uncomplete KD” which overlaps with other forms of febrile Exanthematous illness in children.

In this original paper, Dr. Reyna et al.^4^ highlight the coronary arteries dilatation in the context of febrile Exanthematous illnesses, but not classified as KD.^4^ Interestingly, Kawasaki’s presentations and publication were initially met with skepticism as to whether his cases were a newly recognized disease entity or a variant of scarlet fever, Stevens-Johnson syndrome, or erythema multiforme. So, the most important and key steP is a clear definition and criteria of KD and coronary artery lesions. In recognition of the challenges posed in the diagnosis of “incomplete” KD, The Japanese Ministry of Health Research Committee and the Japanese Circulation Society (JCS), and the American Heart Association (AHA) and American Academy of Pediatrics (AAP ), in 2004, established their criteria.^2,5^ The definitions and criteria for Kawasaki disease diagnosis slightly differ between the AHA/AAP and Japanese guidelines. The diagnostic criteria for classical

Kawasaki disease in AHA/AAP guidelines include fever persisting at least 5 days and at least four of five other criteria. The criteria in the Japanese guidelines include fever as a sixth, equally important criterion, and patients must meet five of six criteria for diagnosis, including fever that subsides within 5 days in response to therapy. It is difficult to compare coronary lesions between these two countries because the definitions of are completely different in the respective guidelines. The Japanese JCS guidelines for Coronary artery lesions use the diameter of each segment of coronary arteries. However, in the AHA/APP guidelines aneurysms are classified using z-scores. In this paper, the author uses echocardiogram to assess the coronary artery luminal dimensions, converted to z - scores adjusted for body surface area (BSA).

As already mentioned before, another important topic related to this publication is the concept of atypical KD, which is very challenging diagnosis and management. The Japanese guidelines state that a KD diagnosis is possible even when five or more of the principal symptoms are absent, if other conditions can be excluded and KD is suspected, a condition known as incomplete KD. Indeed, approximately 15-20% of KD patients have incomplete KD in Japan.^6^ However, even if a patient has four or fewer principal symptoms, the illness should not be regarded as less severe, because cardiovascular abnormalities are not rare in patients with incomplete KD.^7^ The AHA/AAP guidelines include an algorithm for evaluation and treatment of suspected patients with incomplete or atypical KD. The algorithm indicates that incomplete KD should be diagnosed in a patient with a fever persisting at least 5 days, two or three additional clinical diagnostic criteria, and abnormal laboratory values typical of KD. The incidence rate of incomplete KD in the United States is reported to be approximately 20-27%. The AHA/AAP specifies that the term “atypical” should be used to describe patients who have a sign or symptom not typically seen in KD, such as renal impairment.

Previously, in a pilot study Muniz et al.^8^ described that coronary arteries dimensions with non-KD febrile illness are larger than those in normative afebrile subjects but smaller than dimensions in patients with KD.^8^ Some gaps still need to be filled, especially related to the pathology in those febrile illness: in the KD vasculopathy primarily involves muscular arteries and is characterized by 3 linked processes: 1 - necrotizing arteritis; 2 - subacute/chronic vasculitis and 3 - luminal myofibroblastic proliferation. Maybe, a better understanding of this process, which clarifies why the coronary arteries became dilated and don’t progress to aneurysms.

So, the vasculopathy in KD and other febrile exanthemathous illness remains an enigmatic disease. Five decades of new findings and all research have not been enough. We still need more research to give us more answers….
